# Automatic liver segmentation on Computed Tomography using random walkers for treatment planning

**DOI:** 10.17179/excli2016-473

**Published:** 2016-08-10

**Authors:** Mehrdad Moghbel, Syamsiah Mashohor, Rozi Mahmud, M. Iqbal Bin Saripan

**Affiliations:** 1 Department of Computer & Communication Systems, Faculty of Engineering, University Putra Malaysia, 43400 Serdang, Selangor, Malaysia; 2 Cancer Resource & Education Center, University Putra Malaysia, 43400 Serdang, Selangor, Malaysia

**Keywords:** image segmentation, random walker, CT imaging, liver segmentation

## Abstract

Segmentation of the liver from Computed Tomography (CT) volumes plays an important role during the choice of treatment strategies for liver diseases. Despite lots of attention, liver segmentation remains a challenging task due to the lack of visible edges on most boundaries of the liver coupled with high variability of both intensity patterns and anatomical appearances with all these difficulties becoming more prominent in pathological livers. To achieve a more accurate segmentation, a random walker based framework is proposed that can segment contrast-enhanced livers CT images with great accuracy and speed. Based on the location of the right lung lobe, the liver dome is automatically detected thus eliminating the need for manual initialization. The computational requirements are further minimized utilizing rib-caged area segmentation, the liver is then extracted by utilizing random walker method. The proposed method was able to achieve one of the highest accuracies reported in the literature against a mixed healthy and pathological liver dataset compared to other segmentation methods with an overlap error of 4.47 % and dice similarity coefficient of 0.94 while it showed exceptional accuracy on segmenting the pathological livers with an overlap error of 5.95 % and dice similarity coefficient of 0.91.

## Introduction

Liver segmentation is a prerequisite for many clinical and research applications, segmentation of the liver from Computed Tomography (CT) volumes plays an important role in the choice of treatment strategies for liver diseases (Chen et al., 2011[10]). Liver segmentation is difficult as the liver shape is variable and displays low attenuation compared to the neighboring organs, making segmentation of the liver a complex problem. Being a soft organ, liver shape is highly dependent on adjacent organs within the abdomen. Moreover, many pathologies can have a strong effect on the appearance and the shape of the liver while most of the time clearly defined edges are not visible on many sides of the liver. In particular, the intensity difference between the liver and the diaphragm or the spleen or the stomach are very small. Although there are formulas that estimate the liver volume by utilizing the age, weight and the height of the patient, these formulas are highly inaccurate in the case of pathological livers. Joyeux et al. (2003[24]) showed that the correlations between the calculated liver volume by these formulas and the volumes of the liver lobes or anatomical segments in pathological livers were very low.

Despite lots of attention, fully automatic liver segmentation from a CT volume remains a challenging task (Anter et al., 2013[3]), mainly because of the variability of the liver shape and the intensity patterns inside and in the neighborhood of the liver. On the other hand, working inside a liver envelope yields better results in case of segmenting and classifying the lesions inside the liver, previous works have indeed shown that the segmentation of liver lesions was more accurate when done inside liver only, in particular for automatic methods (Taieb et al., 2008[50]; Schmidt et al., 2008[42]; Qi et al., 2008[39]; Ben-Dan and Shenhav, 2008[7]; Shimizu et al., 2008[44]; Smeets et al., 2008[47]; Kubota, 2008[26]; Wong et al., 2008[56]; Moltz et al., 2008[35]; Stawiaski et al., 2008[49]; Zhou et al., 2008[58]). 

To address these concerns, a challenge was presented to different researchers by The Medical Image Computing and Computer Assisted Intervention Society (MICCAI) to segment liver on clinical bases (Heimann et al., 2009[19]). From this challenge, it can be concluded that three main approaches have been proposed and considered for segmentation of the liver envelope namely region growing strategies, probabilistic atlases and statistical shape models.

Region growing strategies are data-driven approaches that iteratively construct a region of interest (ROI) defined at pixel level beginning with an initial set of seeds. Such approaches iteratively cluster neighboring voxels by deciding whether these voxels are to be added to the ROI at any given step. Because of this definition, region growing strategies do not rely on a specific prior model but adapt to each image depending on the seeds given by the user. Despite their simple concept, region growing approaches can still provide satisfactory results compared to more evolved approaches, the method proposed by Rusko et al. (2007[40]) indeed offered one of the best results during the MICCAI segmentation challenge.

Probabilistic atlases utilize both the prior shape and the spatial location information to achieve a refined segmentation. Using probabilistic atlases (PA), Park et al. (2003[38]) segmented the liver by optimizing a Markov random field (MRF) formula dependent on intensity distributions computed through the registration of a PA by thin plates wrapping. Zhou et al. (2008[58]) proposed a segmentation of the liver using a threshold of the probabilities of segmentation being the liver, where this probability is defined at voxel level through PA and an intensity model. Shimizu et al. (2007[45]) proposed a segmentation technique for 12 abdominal organs inside the abdominal cavity, this technique begins with a rough segmentation obtained through PA and priors on intensity distributions inside each organ, utilizing a level-set algorithm for enhancing the final segmentation. Linguraru et al. (2009[32]) proposed a segmentation of pathologic livers and spleens on contrast-enhanced images, an initial segmentation is obtained through series of rigid, affine and nonlinear registrations of one PA, the segmentation is then refined using geodesic active contours and an estimation of the distribution parameters inside the liver. 

Statistical Shape Models (SSM) define a mesh for the liver envelope along with possible deformations of the nodes describing the liver boundary (Heimann et al., 2007[17]). However, the high variability of liver shapes is highly challenging because of the difficulties in defining an SSM that captures the large variations of the liver shapes. Consequently, later authors introduced an additional step in order to improve the limited transformations of SSM. However, the contribution of SSM remains significant as a recent review (Tomoshige et al., 2014[53]) showed that an SSM with a subsequent free deformation step offered one of the best results for automatic liver segmentation. Lamecker et al. (2004[27]) constructed an SSM of the liver that was used for segmentation of livers with lesions using models of gray-level profiles along surface normals to fit the SSM on new images. 

Kainmüller et al. (2007[25]) proposed free forms to define the SSM, this approach proved to be one of the best during the MICCAI segmentation challenge. Heimann et al. (2006[20]) introduced an SSM to segment the liver without major pathologies while mainly following the approach proposed by Lamecker et al. (2004[27]), adding a multi-resolution algorithm and active shape models. Heimann et al. (2007[17][18][21]) later improved his approach with an automatic initialization of the shape model and by replacing the active shape models by a more complex technique. 

Okada et al. (2008[37]) introduced a segmentation approach combining PA and SSM to segment the liver. An initial segmentation is done using PA and then the segmentation is refined with SSM. Ling et al. (2008[31]) proposed a segmentation using a hierarchical shape model, where liver boundaries are detected using learning based approaches. 

In this paper, a segmentation method based on fast random walkers (Andrews et al., 2010[2]) is proposed for segmenting contrast-enhanced CT images in a clinical prospect with potential applications for diagnosis and as a first step for the segmentation of the liver lesions (Moghbel et al., 2016[34]) as the contrast-enhanced CT images remain the main medium for diagnosing liver pathologies. 

## Materials and Methods

**Figure 1:**
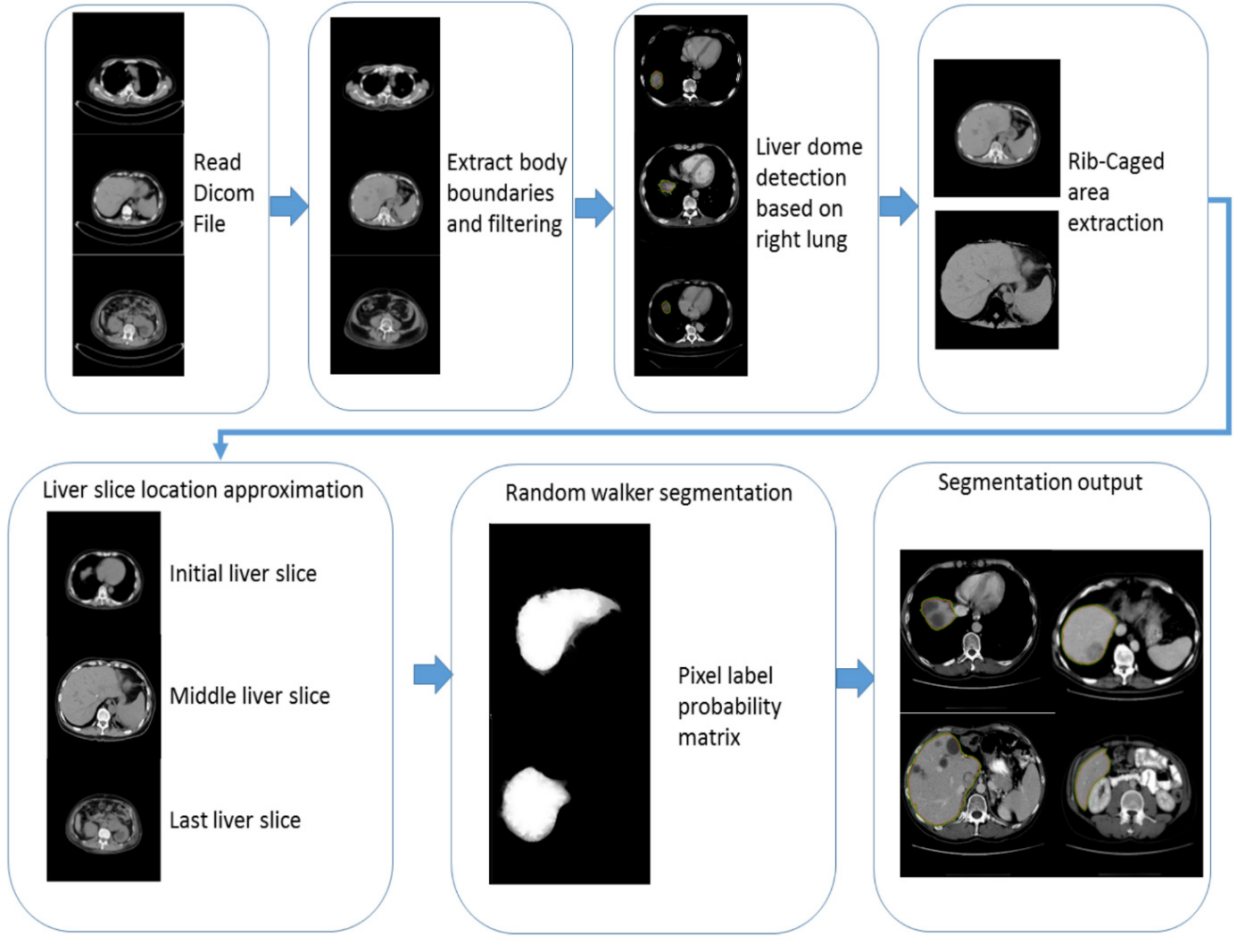
Flowchart of the proposed segmentation

Figure 1(Fig. 1) represents the flowchart of the proposed segmentation algorithm. The detailed workflow of the proposed segmentation framework is discussed in the following sections.

### Background removal and filtering

**Figure 2:**
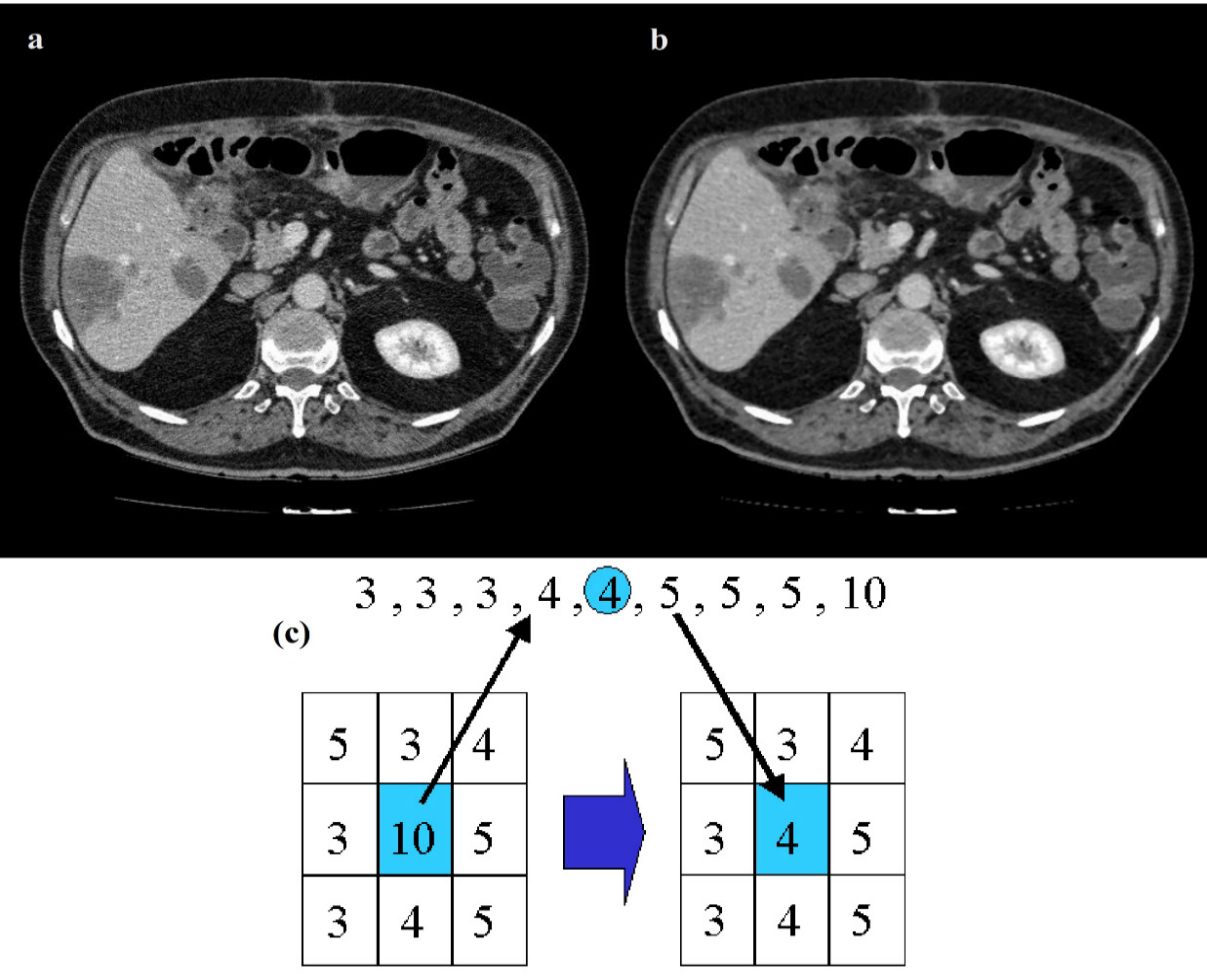
Results of median filtering, original image (a) applied to a CT image (b), median filter (c)

As CT images contain parts of the imaging table along the patient's body, it is required to remove these unwanted portions of the image. To do so, body boundaries are detected by utilizing a Canny edge detector (Russ, 2011[41]). The body is then detected by selecting the morphologically filled region with the largest area in the slice. For increasing the level of edge detail in the image a series of Top-Hat and Bottom-Hat enhancements (Russ, 2011[41]) are applied to the image, Top-hat emphasizes the edges present on the image while the bottom-hat decreases the undesired distortions on the image. The Top-Hat image is added to the original image while the Bottom-Hat image is subtracted from the image. To remove noise in the images a 3×3 median filter is utilized. The main reason median filtering was chosen for the preprocessing step of this algorithm is because median filters have the useful property of retaining edge information within an image as seen in Figure 2(Fig. 2). Mean filters and Gaussian filters tend to blur the edges in an image. This is because the median filter does not create new unrealistic pixel values when the window lays over an edge.

### Liver Dome Detection

**Figure 3:**
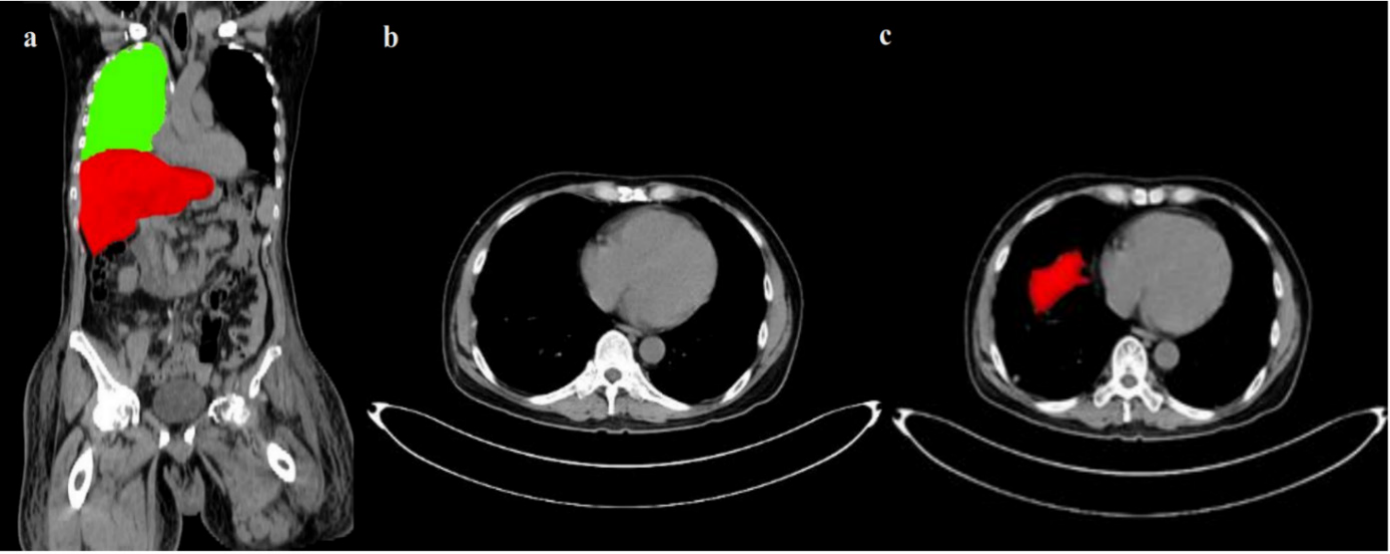
Liver location with respect to lung (green) (a), CT slice just before liver dome (b) detected liver dome in red (c)

As liver sits just below the right lung, initial liver contour (liver dome) can be easily detected by searching from the lung lobe above. As lungs are mostly filled by air and air has the lowest attenuation coefficient in CT images, the lung area is often black resulting in easy detection of the lung lobes with reasonable accuracy. After the middle point of the right lung is detected, if there was a significant decrease (experimentally equal to 10 % on average CT series) in the area of the lung lobe going from superior to the inferior direction in two consecutive slices emergence of the liver dome is implied, as illustrated in Figure 3(Fig. 3). Although the set threshold can result in missing one slice (with a relative small liver dome) in some series, a tradeoff between accuracy and robustness are nevertheless unavoidable.

### Ribcage removal 

**Figure 4:**
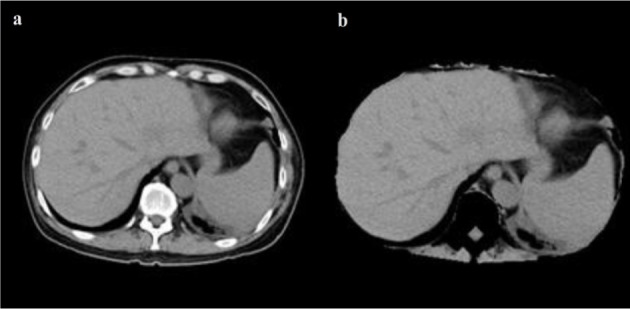
Original CT image (a), CT image after ribcage removal (b)

Since the main body of the liver is protected by the rib cage and to minimize the computational requirements of the segmentation algorithm and in order to remove the bulk of intercostal muscles between ribs, De'Boor algorithm (De Boor, 1972[13]) is utilized for ribcage contour segmentation. The centroid or center point of gravity of each rib in each slice is calculated, then all the points are joined by de Boor's algorithm, to achieve a smooth curve an experimental B-spline order of 3 is utilized. In any axial CT image, especially those towards the caudal direction, there might be slices not containing all the ribs, without enough centroid points a suitable curve enclosing the liver cannot be calculated. To avoid this situation, in case of a steep decrease in the area of segmented CT slice, detected rib centroids in previous and subsequent slices are overlaid on the slice being processed and the B-spline is re-calculated thus avoiding the under segmented slice. Figure 4(Fig. 4) shows a CT slice before and after rib cage removal.

### Proposed segmentation method

Graph-Cut (GC) based segmentation is an alternative to boundary based segmentation methods, being a semi-automatic segmentation the user is required to provide the seeds representing the background and the object to be segmented, GC represents the image pixels as nodes on a graph with weighted edges representing the adjacency between the pixels. By finding the minimum cost function between all possible cuts of the graph, the GC segments the image into background and the object (Boykov et al., 2001[8]). The main disadvantage of regular GC segmentation is the bad handling of weak edges and noisy images, to overcome this limitation many methods have been proposed to enhance the basic GC algorithm. One of such methods receiving a wide interest in medical imaging is the random walker algorithm (Andrews et al., 2010[2]; Cui et al., 2013[12]; Grady and Sinop, 2008[16]). Random walkers segmentation was proposed by Grady (2006[15]), it is a supervised segmentation method meaning that a set of labels must be defined for each object prior to segmentation, this can be done interactively by the operator or be assigned automatically according to a predefined criterion. Random walker method segments the image by calculating the probability


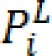


that a random walker starting at pixel '*i'* first reaching a pixel labeled *L*. 

The principle of random walker segmentation is the construction of an undirected graph *G= (V, E)* where the nodes *v* ϵ *V* correspond to image pixels and *e* ∊ *E* ⊑ *V* ⨯ *V*. Weight *W_ij_* is assigned to edge *e_ij_* connecting nodes *v_i_* and *v_j_* based on the following equation: 


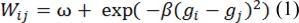


Where *g_i_* is the intensity at the pixel *i* and *g_j_* is the intensity at pixel j. *β* is a scaling parameter set according to image contrast and ω is a regularization parameter that amounts to penalizing the gradient norm of *P^L^* (ω = 0 results in no regularization). In order to increase the performance on complex CT images, the local spatial similarity between local pixels is incorporated into the weighting function, with this addition the weighting function transforms to: 


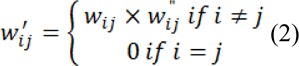


Where the intensity relationship is denoted by *w_ij_* (same as Eq. 1) and local spatial relation


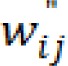


is expressed by:


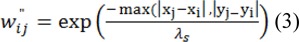


Where (*x_i_, y_i_*) are the spatial coordinates of the *i^th^* pixel and (*x_j_, y_j_*) are the spatial coordinates of the adjacent *j^th^* pixel and *λ_s_* ^represents the scale factor of^


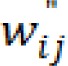


spread. 

Weight *W_ij_* can be described as the probability of the random walker crossing a particular edge, random walkers will cross edges more easily in case of more homogeneous edges created by a lower edge weight and thus region labels are decided more by the pixel distance to seeds labeled *L* and less by image features. Greater values of edge weight create less homogeneous edges thus making it harder for random walkers to cross edges and the region labels are decided more by the locations of strong edges. 

With the help of the circuit theory, Grady (2006[15]) showed that the connections between random walkers on a graph correspond to a combinatorial analog of the Dirichlet problem thus dramatically reducing calculation time by providing a convenient and simple method for the label probabilities computation. 

A Dirichlet problem can be defined as the problem of finding a harmonic function subject to certain boundary values. A Dirichlet integral could be represented as:


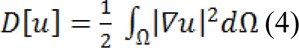


The harmonic function minimizing the Dirichlet integral and satisfying the boundary condition can be achieved by the following Laplace equation:


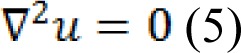


Let's denote *v_m_* as a set of seeded pixels and *v_u_* the set of unseeded pixels, such that *v_u _∩* *v_m = _*∅ and *v_u _*∪ *v_m = _v*. It was shown that all of the probabilities 


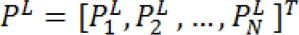


that each node (pixel) *v_i_* ∊ *v_u_* being assigned to label *L *can be obtained with the minimization of:


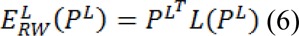


Where the probabilities of seeds P^L^ are assigned as: 


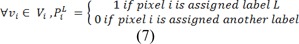


Where combinatorial Laplacian matrix of *L* is defined as:


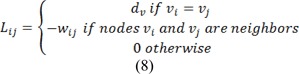


Where *d_v_* is the degree of the vertex of edge *v_i_* (sum of weights of all the edges *e_ij_* connecting *v_i_*), for 2D images the vertices will have a degree of 4 or 8 and for 3D images the vertices could have a degree from 6 up to 26. Eq. 4 can be rewritten as:


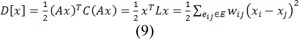


Where *C* is a diagonal matrix edge weights assigned to its diagonal and *A* is the incidence matrix defined as:


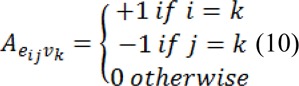


Eq. 9 can be decomposed as:


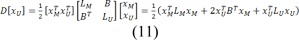


Where *x_U_* represents the probabilities of seeded and *x_M_* represents the probabilities of unseeded nods, critical points are determined by differentiating *D*[*x_U_*] with respect to *x_U_* as:


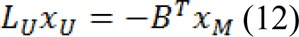


Which represents a system of linear equations where |*L_U_*| represents the unknowns. Using Eq. 7, the combinatorial Dirichlet solution can be found by solving the following equation for all labels:


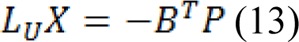


Only *L ˗ 1* systems must be solved as the sum of all probabilities at a node will be equal to zero:


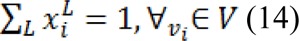


After minimizing


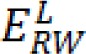


for each label *L*, the segmented region is obtained by calculating maximum probability of the label by: 


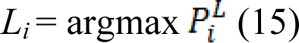


The workflow of the random walker method for image I can be summarized as:

1. Provide a set of marked pixels with L labels corresponding to desired segmentation regions
2. Map the image features such as intensities, texture information or other image features to edge weights and built the Laplacian matrix
3. Perform the random walker and obtain segmentation label for each region.

**Figure 5:**
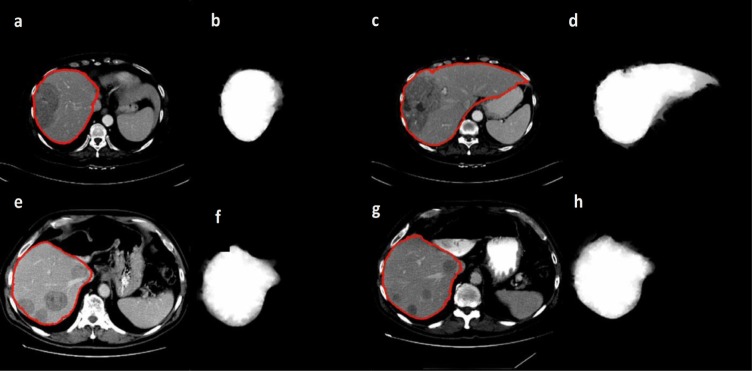
Segmented pathological liver with the random walker algorithm with red line representing the segmentation (a,c,e,g) and the corresponding visualized probability *P^L^_i_* (b,d,f,h) with white representing highest and black representing the lowest probability

In a study comparing different segmentation methods for positron emission tomography (PET) images random walker were found to provide the most accurate results (Bağci et al., 2011[5]), in case of CT images random walker were more accurate than other segmentation methods such as level sets (Chen et al., 2011[9]) and in case of brain MRI random walker performed accurately (Choubey and Agrawal, 2012[11]). Figure 5(Fig. 5) shows segmented pathological livers with the random walker algorithm and the corresponding probability


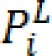
,

it should be noted that the values are pixel specific and are mapped to gray-scale and displayed for easy visualization.

### Seed implementation 

After detection of the liver dome, seeds are implanted automatically according to the position of the liver dome with respect to the image. There are up to a total number of 1200 different seeded pixels for each slice with half of them representing the liver and the other half representing the other organs present in the slice. 

As the liver changes considerably in shape and size, it is desired to have an approximate knowledge of these variations within a series to be able to implement a more robust seeing arrangement. In order to determine the approximate size of the liver, two slices after the detected liver dome are segmented using random walker algorithm. After these slices are segmented, the mean and the deviation of the intensity variation is calculated for these slices, then the right lung mask calculated for the slice just before the liver dome is used as a template and the rest of the CT series are masked based on that. Afterward, the number of the pixels falling within the pre-calculated liver mean and deviation are calculated, the slice with the highest number of pixels falling into the criteria is designated as the middle liver slice (biggest liver slice) and the slice with the lowest number of pixels is designated as the last slice, it should be noted that based on observations, the number of the pixels in the lower parts of the liver are usually around 100 (experimentally derived on standard 512×512 CT images), thus the threshold for detecting the last slice of the liver is set. This middle and last liver slice selection is done in order to have more control over the seeding arrangement and avoiding any overlapping of the seeds.

### Datasets

**Figure 6:**
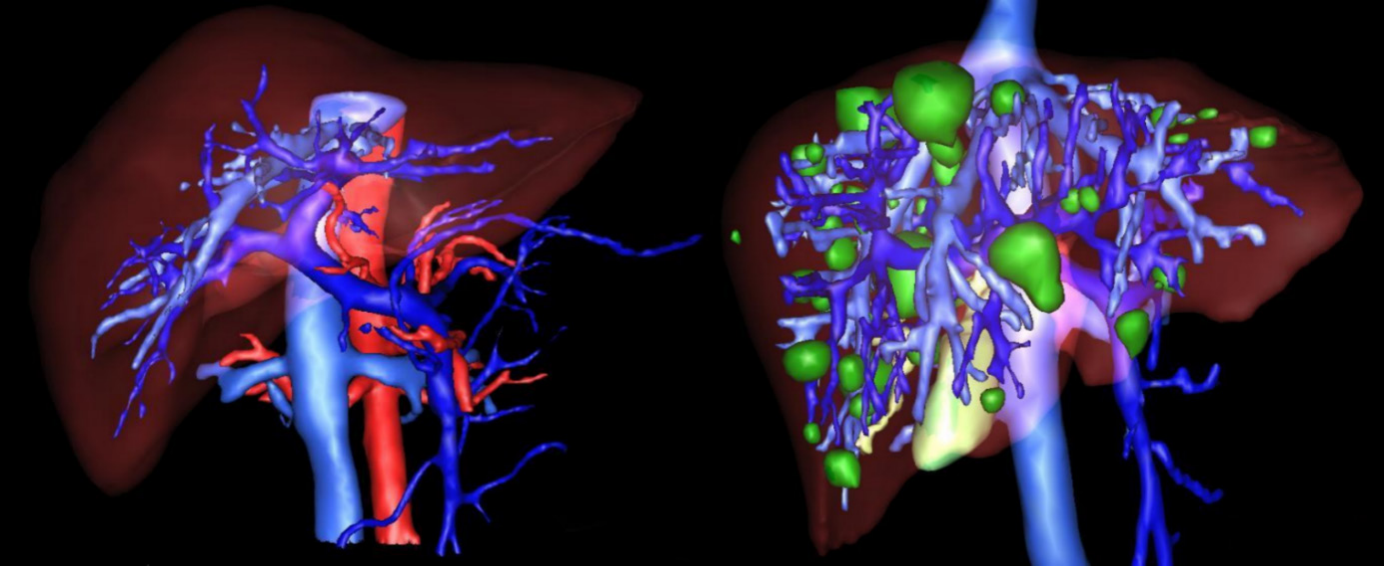
Healthy (left) versus pathological liver (right) from 3Dircadb dataset (lesions represented in green)

Data used in this paper is provided by medical professionals from cancer imaging archive of the Frederick National Laboratory for Cancer Research (TCIA dataset, 2016[51]), 3Dircadb dataset provided by Research Institute against Digestive Cancer (IRCAD dataset, 2016[23]) and The Medical Image Computing and Computer Assisted Intervention Society (MICCAI) liver segmentation challenge (Sliver'07 dataset, 2016[46]). The algorithm is developed on pathological liver images with varying lesions from the cancer imaging archive dataset and then benchmarked against the Sliver'07 and 3Dircadb datasets. It should be noted that the Sliver'07 and 3Dircadb datasets are only used for benchmarking and are not used in the development. The developed framework showed exceptional accuracy in segmenting liver envelope while the typical runtime to segment a series of CT images was around 210 seconds. Only patients with pathologies were utilized from 3Dircadb data set in this study, totaling 15 patients with 120 lesions in total, making segmentation a difficult task and a good measure of segmentation accuracy, Figure 6(Fig. 6) illustrates a pathological case from 3Dircadb dataset.

In case of dataset acquired from the cancer imaging archive, all the patient data is confirmed to belong to cancerous cases by medical experts, while pixel spacing varied from 0.55 to 0.95 mm and slice thickness varied from 1 to 5 mm with all patient identification information removed, it should be noted that only contrast enhanced image data was utilized from this dataset. 

In the case of Sliver'07 and 3Dircadb datasets utilized in this study for liver segmentation benchmarking, radiological experts manually outlined liver contours for all images on a slice-by-slice basis in order to determine the ground truth. The number of slices in each series, the slice thickness and the pixel spacing varied from 64 to 502, 0.5 to 5.0 mm and 0.54 to 0.87 mm respectively. The cases involved few healthy cases, but most of them are pathologic involving metastasis cysts and lesions of different sizes. The image resolution is 512×512 in all cases. 

**Figure 7:**
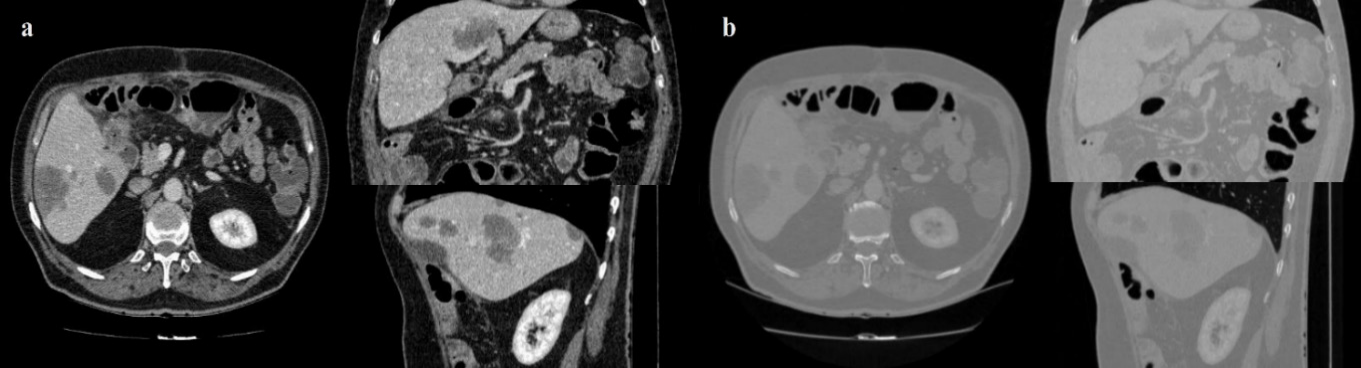
CT image with window level and settings recommendations for Abdominal CT (a), CT image with dynamic window level and settings (b)

All internal structures of the liver such as vessels and lesions are included in the liver mask during manual segmentations. A vessel is considered as a part of liver if it is completely surrounded by liver tissue. If a vessel is partially enclosed by the liver (often the case where large veins-vena cava and portal vein enter or exit the liver), only the parts surrounded by liver tissue are included in segmentation. In the case of Sliver'07 dataset, in order to avoid inconsistency between transversal slices, a binary median filter of 3×3×3 size is performed as a post-processing step while a single expert examined the results and corrected them if necessary. In addition to all that, all patient and center related information in all the datasets were removed prior to making them public. The proposed segmentation is applied to the Sliver'07 and 3Dircadb datasets consisting of 35 series of enhanced CT series. It should be noted that the developed framework was run with Matlab 2013a on a personal computer with 8 GB of ram and an Intel i7 CPU. All the images utilized in this study are processed with a window level and settings recommendations for Abdominal CT imaging as illustrated in Figure 7(Fig. 7). 

### Statistical performance measures 

Before any further discussion on the results, a brief introduction on five statistical performance measures utilized are given below, these statistical measures are commonly used in image segmentation validation. For calculating these statistics we need to consider the following notions:

- True Positive (TP), means region segmented as object that proved to be the object.
- False Positive (FP), means region segmented as object that proved not to be the object.
- False Negative (FN), means region segmented as not object that proved to be the object.
- True Negative (TN), means region segmented as not object that proved not to be the object.

#### Volumetric overlap error 

Volumetric overlap error (VOE) expressed in percent, represents the number of pixels in the intersection of segmented region (A) and the ground truth (B), divided by the number of pixels in the union of A and B. A value of zero represents perfect segmentation while any increase in this value correlates to increased discrepancy between segmentation and ground truth. It can be calculated in percent from the following formula:


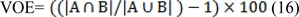


#### Precision or positive predictive value

Precision coefficient represents the overall performance of the algorithm in correctly segmenting the ROI pixels from the image. It can be calculated by the following formula:


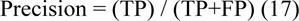


#### Accuracy

Accuracy coefficient represents the overall performance of the algorithm in correctly including the pixels of the ROI inside the segmentation. It can be calculated by the following formula:


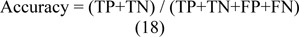


The accuracy of a segmentation system is the degree of closeness of segmentation to the ground truth. The precision of a segmentation system, related to reproducibility and repeatability is the degree to which repeated experiments under unchanged conditions yielding similar results. A measurement system can have high accuracy and low precision or any combinations of those, but a system is considered valid if both precision and accuracy are high.

#### Dice similarity coefficient

Dice similarity coefficient (DSC) also represents the overall performance of the algorithm in correctly including the pixels of the ROI inside the segmentation. It can be calculated by the following formula:


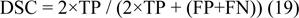


A value of 0 represents no overlap between the segmented region and ground truth while a value of 1 represents perfect segmentation.

#### Relative absolute volume difference

Relative absolute volume difference (RVD) expressed in percent, whereby the total volume of the segmented region is divided by the total volume of ground truth. It can be calculated by the following formula:


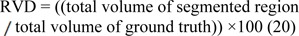


This measure should not be solely utilized to assess the performance of any segmentation method as a value of 0 (perfect segmentation) can also be obtained from an inaccurate segmentation, as long as the segmented region volume is equal to the volume of the ground truth. 

## Results and Discussion

**Figure 8:**
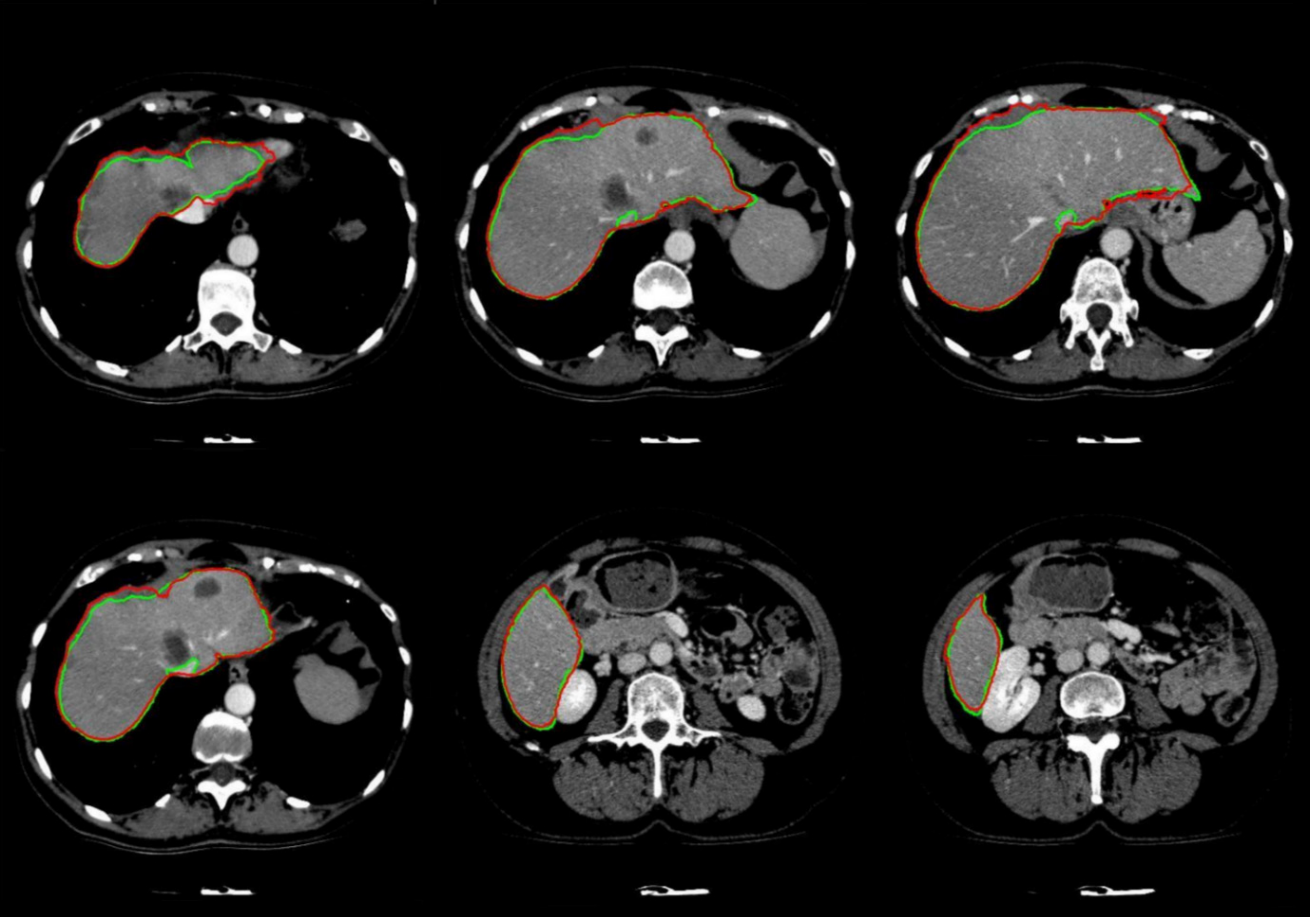
Examples of segmented liver with the proposed framework, with green line representing ground truth and red representing the proposed segmentation

The entire algorithm was able to segment an average series in 3.5 minutes utilizing a typical desktop computer. Average time required to segment a CT series with a slice thickness of 5 mm by an expert is around 30 minutes thus, our proposed method is approximately 9 times faster than manual segmentation while providing acceptable levels of accuracy. Table 1(Tab. 1) represents different statistical performance measures of the developed liver envelope segmentation approach in comparison to expert radiologist segmentation (ground truth) on Sliver'07 train dataset while Table 2(Tab. 2) represents different statistical performance measures of the developed approach in comparison to the expert radiologist segmentation on pathological livers from 3Dircadb dataset, it should be noted that negative values in RVD represent under-segmentation and positive values represent over-segmentation. Figure 8(Fig. 8) illustrates the difference in segmentation by the proposed method on pathological liver slices compared to radiologist segmentation.

From the statistical performance viewpoint, it can be assumed that the developed segmentation framework is amongst the more accurate segmentation methods developed and tested on the liver, achieving comparable result with most semi-automatic methods (Heimann et al., 2009[19]). In medical imaging two of the most accurate measures to ensure robustness and relative performance of the segmentation accuracy are overlap and dice, as these are measured with respect to the performance of the segmentation algorithm in achieving a proper segmentation with respect to the ground truth. In the case of Sliver'07 dataset, the proposed segmentation method was able to achieve an average DSC of 0.94 while the VOE is at 4.47 %, resulting a good overall average accuracy in extracting the liver envelope. As discussed earlier a segmentation method is considered valid if both precision and accuracy are high, the proposed method was able to achieve an average 0.95 and 0.99 in precision and accuracy respectively, clearly collaborating with the result of Dice Similarity Coefficient. Average DSC of 0.91 and VOE of 5.95 % was the performance of the proposed segmentation algorithm on pathological cases over the 3Dircadb dataset with an average 0.92 and 0.99 in precision and accuracy respectively. Considering that the 3Dircadb dataset consisted of many lesions making automatic segmentation considerably more difficult, these results are very promising. Table 3(Tab. 3) (References in Table 3: Maklad et al., 2013[33]; Beichel et al., 2007[6]; Goryawala et al., 2014[14]; Li et al., 2014[29]; Song et al., 2014[48]; Mostafa et al., 2015[36]; Shi et al., 2015[43]; Kainmüller et al., 2007[25]; Al-Shaikhli et al., 2015[1]; Wang et al., 2015[54]; Huang et al., 2014[22]; Xu et al., 2015[57]; Li et al., 2014[28]; Anter et al., 2014[4]; Wang et al., 2015[55]; Heimann et al., 2009[19]) compares the developed method with some of the more accurate methods proposed for liver segmentation. 

While preparing the MICCAI challenge (Heimann et al., 2009[19]), the organizers observed that liver VOE of 6.4 % was the average performance of human segmentation (with medical training) compared to ground truth, thus it can be assumed that the performance of a method with an error rate equal or smaller than the mentioned 6.4 % is comparable to a human operator. 

With a mean VOE of 4.47 % on mixed Sliver'07 dataset used by most other methods in the literature, the proposed segmentation method is comparable to other developed methods while the runtime of around 210-second means that the proposed method is viable as an alternative approach to manual segmentation by a radiologist. As discussed earlier, segmentation of segmentation of pathological livers is much more desirable. The proposed method was able to achieve a mean VOE of 5.95 % and a mean DSC of 0.91 on the very challenging 3Dircadb dataset while the other method that based its segmentation on pathological livers was able to achieve a mean DSC of 0.92 by a semi-automatic approach on a private dataset. Based on these results it can be assumed that the proposed method is comparable to human segmentation performance. Compared to most other segmentation approaches, our approach has the advantage of calculating the rib-caged area thus reducing the possibility of segmentation leakage and the inclusion of the muscle tissue as the liver, thus increasing the segmentation accuracy.

Although the relative volume difference (RVD) calculation and determining the liver volume is of importance as discussed earlier, segmentation algorithms still can achieve a high score in this area while being quite inaccurate as the algorithm can still give accurate volume calculations while the segmented region is widely inaccurate compared to the ground truth. This can be also observed from the overlap error of different segmentation methods, as a method with high overlap error can have a low relative volume difference error. Mean RVD of 2.38 % and 7.49 % has been achieved for Sliver'07 and 3Dircadb datasets respectively, making the proposed segmentation method comparable with other segmentation methods proposed. 

**Figure 9:**
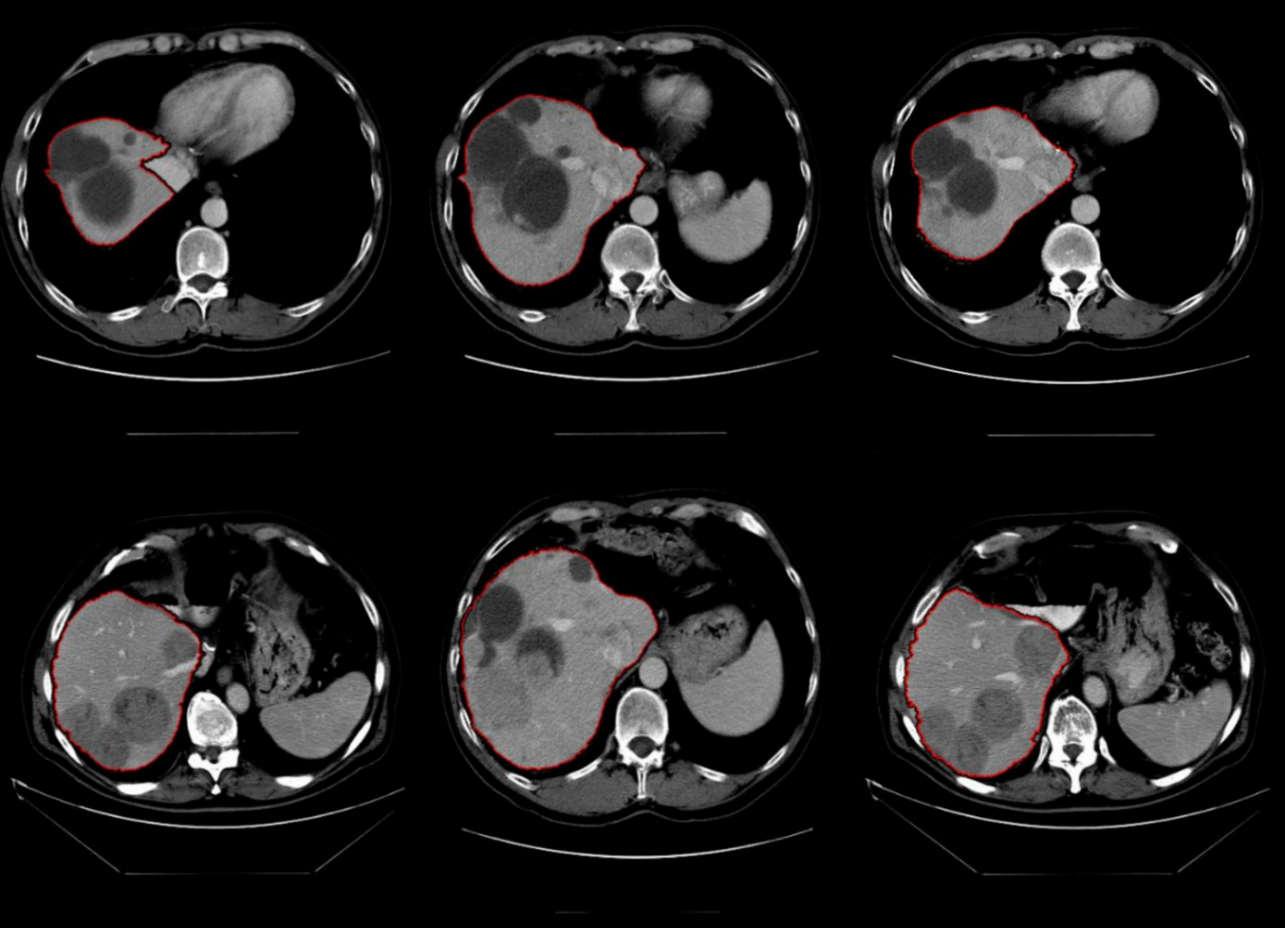
Examples of difficult cases with lesions on liver border segmented using random walker algorithm

The use of liver location estimation based on lungs effectively removes the need for any models to estimate the location of liver, enabling the random walker segmentation to segment the liver more accurately. In case any lesions are present inside the liver envelope, these lesions are also seeded and the ability of multi-region segmentation of the random walker means that these lesions will be also segmented with no regards to their location inside the liver, as segmenting the pathological livers in contrast-enhanced CT imaging are the main reason for taking the challenge of liver segmentation. Examples of challenging pathological livers segmented can be seen in Figure 9(Fig. 9). 

These examples represent severe pathological disorders on livers that make segmentation a very challenging task compared to the data utilized in Sliver'07 challenge, as it can be seen the proposed segmentation algorithm was able to achieve satisfactory results. The main strength of the random walker algorithm compared to most other segmentation approaches is its ability to segment pathological livers accurately and within an acceptable time frame as the main goal of most liver segmentation methods is to increase the speed and the accuracy of liver lesion segmentation. The proposed method require no training thus removing the influence of different datasets on each other as segmentation on each slice is dependent on the liver segmentation from the previous slice compared to model-based and learning-based segmentations where pathological livers with large lesions represent the main disadvantage (Li et al., 2015[30]; Tibamoso and Rueda, 2009[52]; Rusko et al., 2007[40]; Tomoshige et al., 2014[53]; Okada et al., 2008[37]).

**Figure 10:**
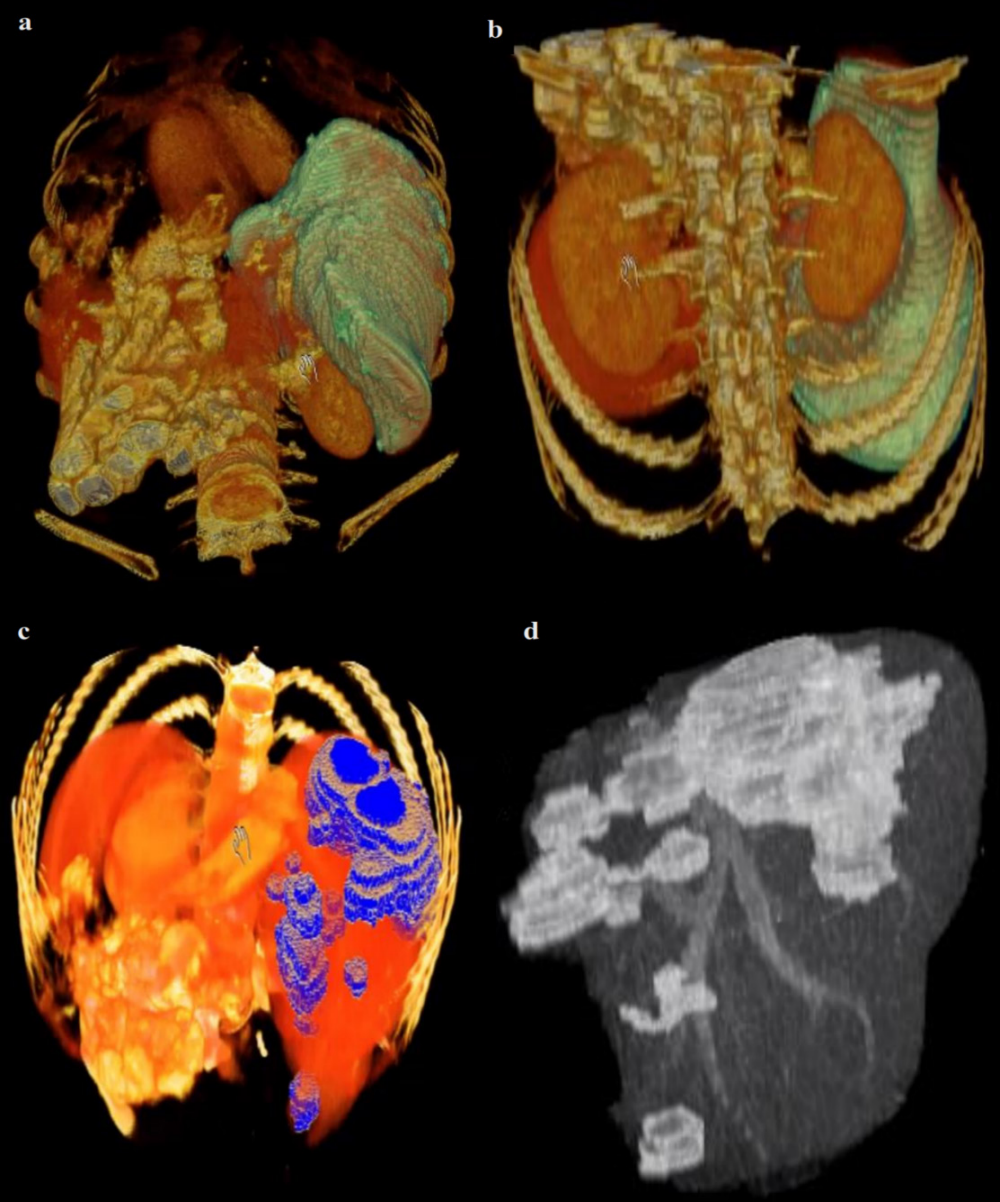
Example of 3D representation of a segmented liver by the proposed method (a,b) (liver in light green) and the segmented liver with visible lesions (c,d)

After the liver is extracted from the CT series, 3D virtualization can be utilized to help the physician in better visualizing the liver and possible lesions inside the liver, this is done as going through a CT series on a slice by slice basis is both tedious and time-consuming. Figure 10(Fig. 10) represents the 3D reconstruction of a segmented liver with pathologies. 

## Conclusions

Proper segmentation of liver envelope is the basis for any accurate CAD system utilized in lesion segmentation and classification while it is the basis of any surgery planning as accurate volume calculation and liver location visualization is the key in accurate prognosis. In this paper, random walker based segmentation for the liver envelope is proposed with the main goal of segmenting pathological livers in contrast-enhanced CT images. The proposed framework was able to achieve a mean VOE of 4.47 % and DSC of 0.94 on Sliver'07 dataset resulting in a performance comparable with human segmentation while in the case of pathological livers from 3Dircadb dataset, the mean overlap error was 5.9 % while DSC was 0.91. The proposed method is amongst the more accurate segmentation frameworks developed for liver envelope segmentation. Random walker has proved to be one of the most accurate segmentation approaches especially for medical imaging and can provide accurate segmentation in livers containing multiple lesions. Main disadvantage of the proposed method lays in the liver dome detection and last liver slice detection as some information might get lost as the algorithm might not consider some CT slices on top and bottom of the liver, although this affects final volume calculation minimally, further work can be done in order to enhance this selection of slices. The developed framework can also be easily applied to other organs within abdomen such as spleen, lungs, aorta and kidneys. 

## Conflict of interest

Authors declare that they have no conflict of interest.

## Figures and Tables

**Table 1:**
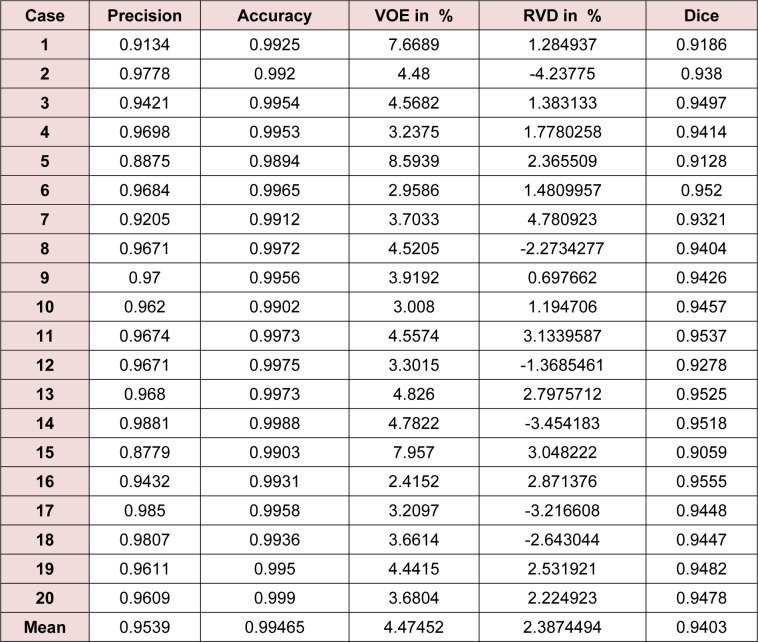
Statistical performance of the developed liver envelope segmentation approach in comparison to ground truth on Sliver'07 dataset

**Table 2:**
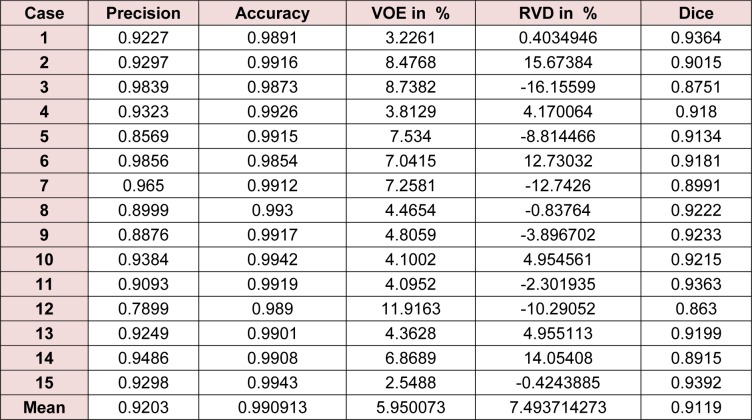
Statistical performance of the developed liver envelope segmentation approach in comparison to expert radiologist segmentation on 3Dircadb dataset

**Table 3:**
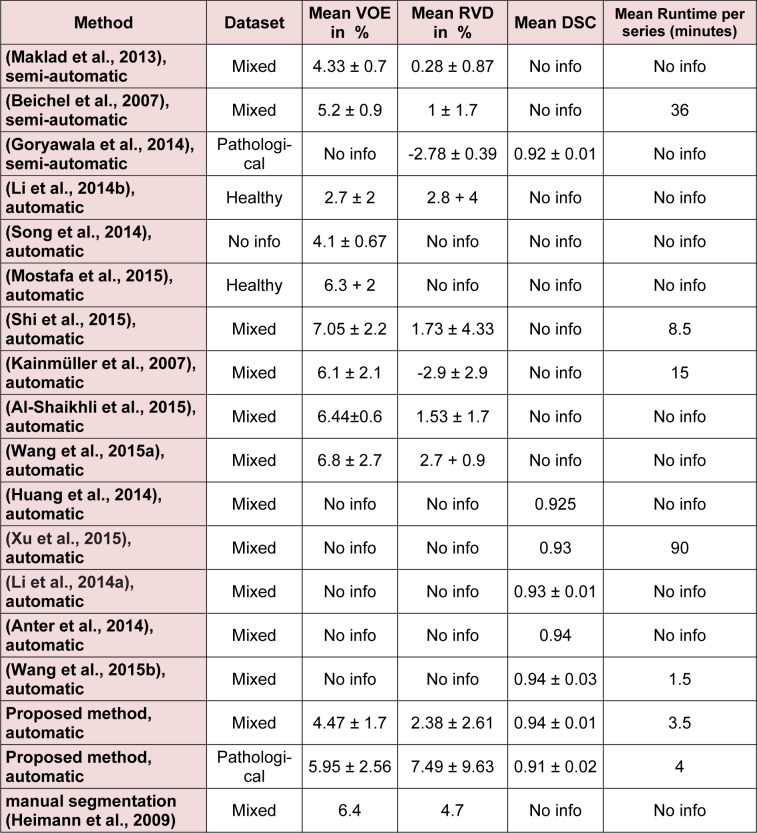
Comparison between the developed method and some of the more accurate methods published on liver segmentation
